# Prolonged PR interval and incidence of atrial fibrillation, heart failure admissions, and mortality in patients with implanted cardiac devices: A real-world survey

**DOI:** 10.1016/j.hroo.2022.12.009

**Published:** 2022-12-22

**Authors:** Hirad Yarmohammadi, Elaine Y. Wan, Angelo Biviano, Hasan Garan, Jodi L. Koehler, Robert W. Stadler

**Affiliations:** ∗ Division of Cardiology, Department of Medicine, Columbia University Vagelos College of Physicians and Surgeons, New York, New York; † Medtronic plc, Mounds View, Minnesota

**Keywords:** PR interval, Atrial fibrillation, Heart failure, Clinical outcomes, Pacemaker

## Abstract

**Background:**

Prolongation of the PR interval has long been considered a benign condition, particularly in the setting of nonstructural heart disease.

**Objective:**

The purpose of this study was to investigate the effect of PR interval on various well-adjudicated cardiovascular outcomes using a large real-world population data of patients with implanted dual-chamber permanent pacemakers or implantable cardioverter-defibrillators.

**Methods:**

PR intervals were measured during remote transmissions in patients with implanted permanent pacemakers or implantable cardioverter-defibrillators. Study endpoints (time to the first occurrence of AF, heart failure hospitalization [HFH], or death) were obtained between January 2007 and June 2019 from the deidentified Optum de-identified Electronic Health Record dataset.

**Results:**

A total of 25,752 patients (age 69.3 ± 13.9 years; 58% male) were evaluated. The average intrinsic PR interval was 185 ± 55 ms. In the subset of 16,730 patients with available long-term device diagnostic data, a total of 2555 (15.3%) individuals developed AF during 2.59 ± 2.18 years of follow-up. The incidence of AF was significantly higher (up to 30%) in patients with a longer PR interval (ie, PR interval ≥270 ms; *P <* .05). Time-to-event survival analysis and multivariable analysis showed that PR interval ≥190 ms was significantly associated with higher incidence of AF, HFH, or HFH or death when compared with shorter PR intervals (*P <* .05 for all 3 parameters).

**Conclusion:**

In a large real-world population of patients with implanted devices, PR interval prolongation was significantly associated with increased incidence of AF, HFH, or death.

**Key Findings**

▪ In a large real-world population of patients with implanted devices, PR interval prolongation was significantly associated with increased incidence of atrial fibrillation, heart failure hospitalization, or death.
▪ Specifically, there was a significant increase in incidence of atrial fibrillation, heart failure hospitalization, or death with an intrinsic PR interval ≥190 ms.
▪ These data suggest that patients might benefit from shortening of PR interval duration using physiological cardiac pacing to reduce the cardiovascular morbidities associated with a prolonged PR interval.

## Introduction

The PR interval measures the time from the beginning of atrial depolarization to the onset of ventricular depolarization.^1^ A prolonged PR interval defined as a PR interval >200 ms, also known as first-degree atrioventricular (AV) block, is commonly seen in the general population, with higher prevalence in older individuals.^2^ The prevalence of a prolonged PR interval across different studies ranged from 2% to 14%, with overall mean prevalence of 7%.^3^

Initially, a prolonged PR interval was considered to be a benign condition.^4^ However, multiple studies have noted that PR interval prolongation can be associated with an increase in incidence of pacemaker implantation, atrial fibrillation (AF), heart failure (HF), and all-cause mortality.^5^, ^6^, ^7^, ^8^ Yet it is not clear how mechanistically PR interval prolongation is associated with various detrimental cardiovascular outcomes. It is possible that the prolonged PR interval either is a marker of more severe conduction and cardiovascular disease or is itself contributing to worsening cardiac function via AV dyssynchrony, diastolic mitral regurgitations, and other possible mechanisms.^8^^,^^9^ Currently, based on the American Heart Association/American College of Cardiology/Heart Rhythm Society guidelines, pacemaker implantation is only indicated if patients with a prolonged PR interval are symptomatic or have neuromuscular disease such as myotonic dystrophy.^10^^,^^11^ Meanwhile, patients with a prolonged PR interval, sinus node dysfunction, and preserved ejection fraction undergoing pacemaker implantation are not currently a group indicated to receive synchronized, His, or septal left bundle pacing as a standard of care.

The purpose of this study was to investigate the effect of the PR interval on various well-adjudicated cardiovascular outcomes using a large real-world population data of patients with implanted dual-chamber permanent pacemakers (PPMs) or implantable cardioverter-defibrillators (ICDs). In addition, we sought to identify the specific threshold of the PR interval that correlates with the adverse outcomes.

## Methods

### Study design

**Figure 1:**
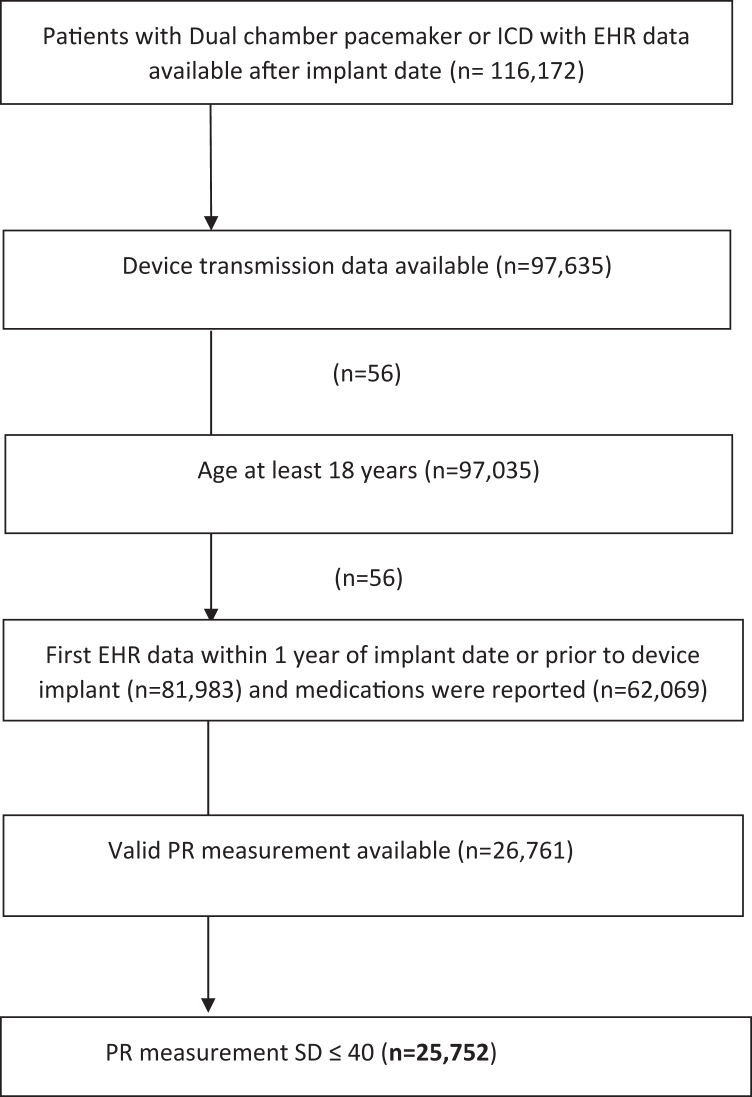
Flow chart of patient selection. EHR = electronic health record; ICD = implantable cardioverter-defibrillator.

The data were extracted from the remote transmissions collected for the Medtronic CareLink (Medtronic, Minneapolis, MN) and Optum de-identified Electronic Health Record dataset. All patient identifiers were removed to comply with HIPAA regulations, and the patients were from the centers who had received consent for the deidentified data use. Based on prior publications, using the de-identified Medtronic CareLink data falls into the category of nonhuman research; therefore, no Institutional Review Board approval was indicated for use of these deidentified data.^12^ The method has been described in previous studies.^13^^,^^14^ A total of 116,172 patients who received dual-chamber PPMs or ICDs between January 2007 and June 2019 were screened. Patients with no transmissions, with missing atrial sensing data, and who were younger than 18 years of age were excluded. Finally, we excluded patients with missing PR interval or inconsistent PR intervals (ie, SD ≤40 ms) to arrive at the final cohort of the patients (Figure 1).

### PR interval measurement

The intrinsic PR interval was measured between atrial and ventricular sense markers from the 10-second CareLink presenting rhythm strip obtained from each patient within the first 30 days of device implantation. A valid PR interval was defined as at least 1 transmitted 10-second strip with at least 3 PR intervals with a single atrial sense between 2 ventricular sensed events. The standard deviation of all valid PR intervals had to be ≤40 ms for inclusion. The PR intervals of the overall cohort were divided into quintiles to determine the incidence of clinical outcomes.

### Outcomes

The endpoints for this study were time to the first occurrence of AF, HF hospitalization (HFH), or death. Device-detected persistent AF was defined as at least 23 hours of AF in a single day, in which the atrial rate exceeded a nominal threshold of 171 beats/min with evidence of 2:1 or greater AV conduction. The burden of AF was then calculated as a summation of these events.^15^ Only patients with available cardiac compass data (16,730 individuals) were included in the assessment of AF outcome. HFH and mortality (all-cause mortality) data were extracted from the Optum de-identified Electronic Health Record dataset. HF events were defined as inpatient, observation unit, or emergency room stay with primary diagnosis of HF and intravenous diuresis administration.

### Statistical analysis

Continuous variables are presented as mean ± SD. Categorical variables are reported as frequency and percentage. For time-to-event survival analyses, cumulative probabilities of the risk of incident AF, HFH, or death were displayed according to the Kaplan-Meier (KM) method, with comparison of cumulative event rates by the log-rank test (time 0 was the maximum of device implantation date and first electronic health record date). Cox proportional hazards regression analyses were performed for multivariable survival analyses of the association between the PR interval and the outcomes. Analyses were adjusted for confounders such as age, sex, prior beta-blocker or antiarrhythmic use, ventricular pacing >40%, and type of implanted device. A 2-sided *P* value of <.05 was considered statistically significant. Statistical analyses were performed using SAS version 9.4 statistical software (SAS Institute, Cary, NC).

## Results

The study consisted of 25,752 patients with implanted dual-chamber PPMs or ICDs with mean age of 69.3 ± 13.9 years (58% male). A total of 19% of the patients had a history of myocardial infarction and 43% had a history of coronary artery disease. A total of 62% of patients had PPMs and 38% had ICDs. The average intrinsic PR interval was 185 ± 55 ms. The mean follow-up was 2.99 ± 2.31 years. The baseline demographics and characteristics of the patients are shown in Table 1. During the follow-up, 2555 (15.3% of 16,730 patients with cardiac compass data) patients developed AF, 2211 (8.6% of 25,752) had HFH and 2264 (8.8% of 25,752) of the patients died. There were only 74 devices in our dataset of 25,752 (0.3%) that were suspected to have conduction system pacing (ie, they had a 3830-69 lead with right ventricular chamber listed).

**Table 1:** Demographic and characteristics of dual-chamber PPM and ICD

Age, y	69.3 ± 13.9
Male	14,842 (58)
Hypertension	14,802 (57)
Heart failure	6960 (27)
Diabetes	6171 (24)
CAD	11,103 (43)
MI	4884 (19)
Vascular disease	2308 (9)
Atrial fibrillation	6710 (26)
Renal dysfunction	3793 (15)
Stroke/TIA	4526 (18)
Device type	
ICD	9792 (38)
Pacemaker	15,960 (62)
Medications	
ACE inhibitor/ARB	13,169 (51)
Beta-blocker	12,461 (48)
Diuretic	11,406 (44)
Spirinolactone	2769 (11)
Entresto	69 (0.3)
Vasodilator/nitrate	11,308 (44)
AAD	17,248 (67)
Anticoagulation	7615 (30)

Values are mean ± SD or n (%).

AAD = antiarrhythmic drug; ACE = angiotensin-converting enzyme; ARB = angiotensin receptor blocker; CAD = coronary artery disease; ICD = implantable cardioverter-defibrillator; MI = myocardial infarction; PPM = permanent pacemaker; TIA = transient ischemic attack.

### PR prolongation and incidence of AF

**Figure 2:**
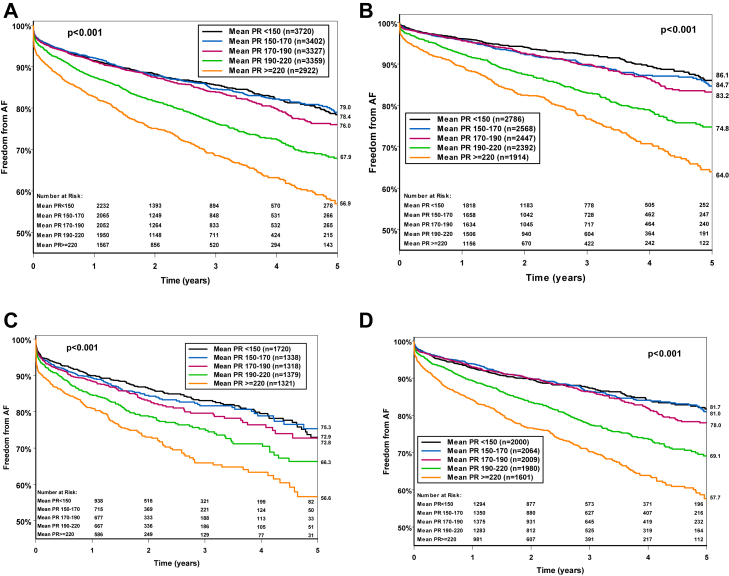
Unadjusted Kaplan-Meier estimates of freedom from atrial fibrillation (AF) among increasing quantiles of PR interval—both permanent pacemaker (PPM) and implantable cardioverter-defibrillator (ICD) in all patients **(A)**, in patients with no history of pre-existing AF in both PPM and ICD **(B)**, PPM only **(C)**, and ICD only **(D)**—with quantile 1 (mean PR interval <150 ms; black line), quantile 2 (mean PR interval 150–170 ms; blue line), quantile 3 (mean PR interval 170–190 ms; purple line), quantile 4 (mean PR interval 190–220 ms; green line), and quantile 5 (mean PR interval ≥220 ms; orange line) shown over 5 years of follow-up. Log-rank test for comparison, *P* < .001.

The cumulative percentage of freedom from AF according to quintiles of the baseline PR interval is shown in Figure 2A in all patients. Figure 2B shows the cumulative percentage of freedom from AF according to quintiles of the baseline PR interval after excluding patients with pre-existing history of AF. AF was significantly more prevalent in the quintiles with PR interval ≥220 ms and PR interval 190 to 220 ms when compared with shorter PR intervals (*P <* .001) in all patients as well as in those with no pre-existing history of AF (Figures 2A and 2B). The same patterns were noted when the freedom from AF was evaluated separately in patients with PPM or ICD (Figures 2C and 2D). The KM curve started to diverge with in the first year of follow-up and continued thereafter.

In multivariable analysis, age, sex (male), prior use of beta-blockers, ventricular pacing >40%, and type of device (PPM) were associated with higher incidence of AF. After adjustment for all confounders, PR interval ≥190 ms was significantly associated with incident AF (Table 2).

**Table 2:** Multivariable Cox proportional hazards models

Variable	Model for AF		Model for HFH		Model HFH/death	
	HR (95% CI)	*P* value	HR (95% CI)	*P* value	HR (95% CI)	*P* value
Age	1.020 (1.016–1.023)	<.001∗	1.012 (1.01–1.016)	<.001∗	1.027 (1.024–1.030)	<.001∗
Female	0.90 (0.82-0.98)	.015∗	1.26 (1.13–1.40)	<.001∗	1.06 (0.98–1.16)	.141
Prior BB use	1.5 (1.38–1.63)	<.001∗	1.31 (1.18–1.46)	<.001∗	1.30 (1.20–1.41)	<.001∗
Prior AAD use	0.98 (0.89–1.07)	.639	1.21 (1.08–1.36)	.001∗	1.07 (0.99–1.17)	.098
VP >40%	1.36 (1.19–1.54)	<.001∗	1.08 (0.87–1.32)	.489	1.00 (0.86–1.17)	.998
AF	NA	NA	1.55 (1.38–1.74)	<.001∗	1.36 (1.24–1.49)	<.001∗
ICD device	0.83 (0.77-0.91)	<.001∗	2.64 (2.30–3.02)	<.001∗	2.09 (1.90–2.30)	<.001∗
Mean PR interval		<.001∗		<.001∗		<.001∗
<150 ms	Reference group		Reference group		Reference group	
150–170 ms	0.97 (0.84–1.11)		1.07 (0.91–1.27)		1.03 (0.91–1.16)	
170–190 ms	1.05 (0.92–1.20)		1.17 (0.99–1.37)		1.08 (0.96–1.22)	
190–220 ms	1.40 (1.23–1.59)∗		1.28 (1.09–1.51)∗		1.19 (1.06–1.34)∗	
≥220 ms	1.80 (1.58–2.04)∗		1.65 (1.41–1.95)∗		1.36 (1.20–1.53)∗	

AAD = antiarrhythmic drug; AF = atrial fibrillation; BB = beta-blocker; CI = confidence interval; HFH = heart failure hospitalization; HR = hazard ratio; ICD = implantable cardioverter-defibrillator; NA = not applicable; VP = ventricular pacing.

∗ Statistical significant.

### PR prolongation and incidence of HFH

**Figure 3:**
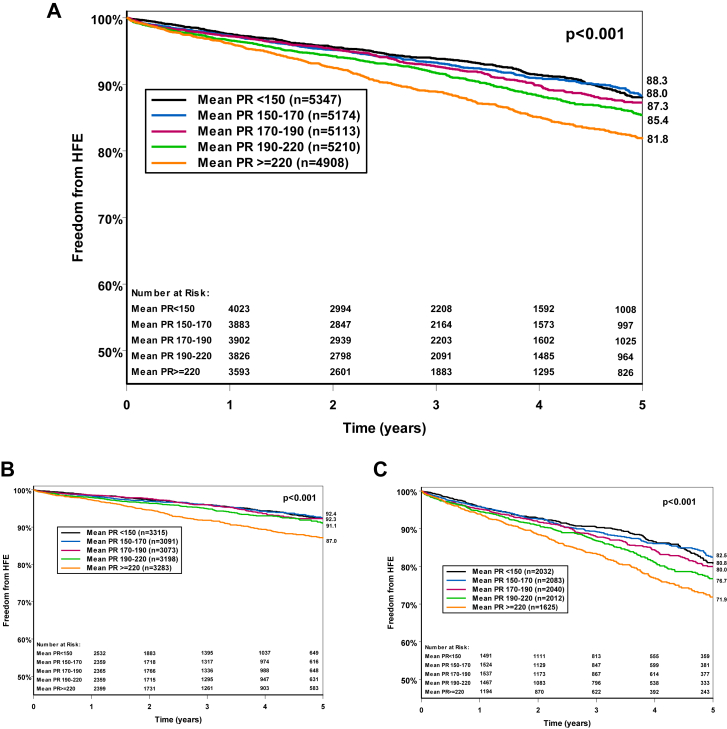
Unadjusted Kaplan-Meier estimates of freedom from heart failure hospitalization among increasing quantiles of PR interval—both permanent pacemaker and implantable cardioverter-defibrillator **(A)**, permanent pacemaker only **(B)**, and implantable cardioverter-defibrillator only **(C)**—with quantile 1 (mean PR interval <150 ms; black line), quantile 2 (mean PR interval 150–170 ms; blue line), quantile 3 (mean PR interval 170–190 ms; purple line), quantile 4, (mean PR interval 190–220 ms; green line), and quantile 5 (mean PR interval ≥220 ms; orange line) shown over 5 years of follow-up. Log-rank test for comparison, *P* < .001. HFE = hospitalization for heart failure exacerbation.

The cumulative percentage of freedom from HFH according to quintiles of the baseline PR interval is shown in Figure 3A. HFH was significantly more likely in the quintile with PR interval ≥220 ms and PR interval 190 to 220 ms when compared with shorter PR intervals (*P <* .05). The same patterns were noted when the freedom from HFH was evaluated separately in patients with PPMs or ICDs (Figures 3B and 3C). The KM curve started to diverge gradually especially after the second year of the follow-up. As expected, HFH was higher in patients with ICDs compared with those with PPMs.

In multivariable analysis, age, sex (female), prior use of beta-blockers, prior use of antiarrhythmics, history of AF, and type of device (ICD) were associated with higher incidence of HFH. After adjustment for all confounders, PR interval ≥190 ms was significantly associated with HFH (Table 2).

### PR prolongation and incidence of HFH or death

**Figure 4:**
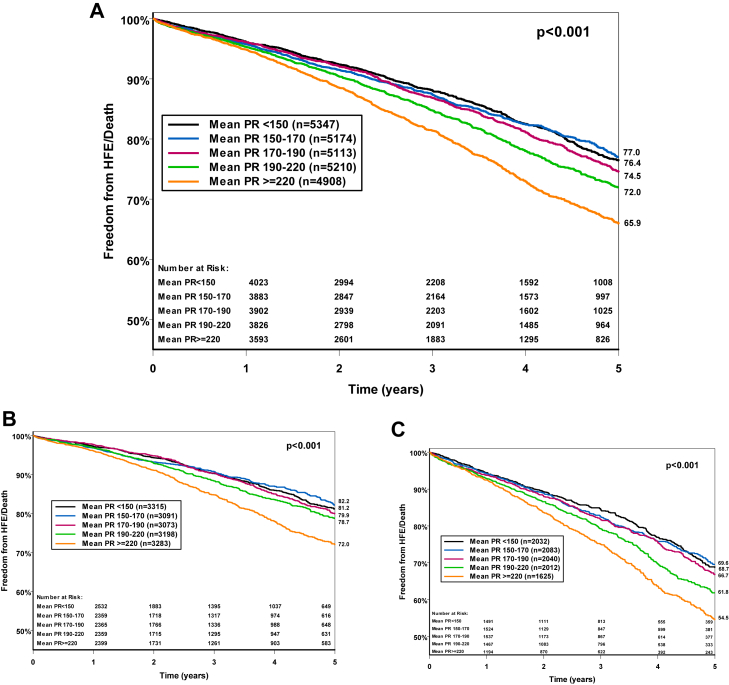
Unadjusted Kaplan-Meier estimates of freedom from heart failure hospitalization or death among increasing quantiles of PR interval—both permanent pacemaker and implantable cardioverter-defibrillator **(A)**, permanent pacemaker only **(B)**, and implantable cardioverter-defibrillator only **(C)**—with quantile 1 (mean PR interval <150 ms; black line), quantile 2 (mean PR interval 150–170 ms; blue line), quantile 3 (mean PR interval 170–190 ms; purple line), quantile 4 (mean PR interval 190–220 ms; green line), and quantile 5 (mean PR interval ≥220 ms; orange line) shown over 5 years of follow-up. Log-rank test for comparison, *P* < .001. HFE = hospitalization for heart failure exacerbation.

The cumulative percentage of freedom from combined HFH or death according to quintiles of the baseline PR interval is shown in Figure 4A. HFH or death was significantly more likely in the quintile with PR interval ≥220 ms and PR interval 190 to 220 ms when compared with shorter PR intervals (*P <* .001). The same patterns were noted when the freedom from HFH or death was evaluated separately in patients with PPMs or ICDs (Figures 4B and 4C). The KM curve started to diverge gradually especially after the second year of the follow-up. In multivariable analysis, age, prior use of beta-blockers, history of AF, and type of device (ICD) were associated with higher incidence of HFH or death. After adjustment for all confounders, PR interval ≥190 ms was significantly associated with incident of HFH or death (Table 2).

When PR intervals were divided to quintiles with 10-ms range, consistent increase in trend of incident AF, HFH, or death was associated with worsening PR intervals (Table 3). In addition, slightly higher percentage of patients with AF and HFH or death was noted for the shortest PR interval (≤130 ms) when compared with longer PR intervals.

**Table 3:** PR intervals in 10-ms quantiles and outcomes

Mean PR interval	Subjects	HFE	HFE/death	Subjects with AF data available∗	AF
≤130 ms	2507	165 (6.58)	392 (15.64)	1691	218 (12.89)
130–140 ms	1518	129 (8.50)	219 (14.43)	1170	121 (10.34)
140–150 ms	2047	153 (7.47)	288 (14.07)	1520	167 (10.99)
150–160 ms	2702	178 (6.59)	394 (14.58)	1695	203 (11.98)
160–170 ms	2570	214 (8.33)	422 (16.42)	1793	204 (11.38)
170–180 ms	2430	214 (8.81)	434 (17.86)	1671	216 (12.93)
180–190 ms	2569	201 (7.82)	434 (16.89)	1493	227 (15.20)
190–200 ms	1831	161 (8.79)	328 (17.91)	1231	203 (16.49)
200–210 ms	1672	139 (8.31)	303 (18.12)	1038	200 (19.27)
210–220 ms	1319	132 (10.01)	258 (19.56)	780	146 (18.72)
220–230 ms	941	103 (10.95)	187 (19.87)	566	116 (20.49)
230–240 ms	833	94 (11.28)	163 (19.57)	446	104 (23.32)
240–250 ms	606	79 (13.04)	150 (24.75)	324	68 (20.99)
250–260 ms	433	49 (11.32)	93 (21.48)	283	66 (23.32)
260–270 ms	385	30 (7.79)	81 (21.04)	193	57 (29.53)
270–280 ms	274	33 (12.04)	62 (22.63)	146	48 (32.88)
280–290 ms	204	25 (12.25)	45 (22.06)	119	34 (28.57)
290–300 ms	162	25 (15.43)	46 (28.40)	100	27 (27.00)
>300 ms	749	87 (11.62)	176 (23.50)	471	130 (27.60)

Values are n or n (%).

AF = atrial fibrillation; HFE = hospitalization for heart failure exacerbation.

∗ Only 16,730 subjects had cardiac compass data available to evaluate the AF endpoint.

## Discussion

This study sought to evaluate the long-term cardiovascular outcomes of a prolonged PR interval in patients with implanted PPMs or ICDs. The investigation was based on a contemporary, community-based cohort, with standardized assessment of baseline risk factors and outcomes, including arrhythmic endpoints. The study demonstrated a significant 20% to 80% increase in incidence of AF, HFH, and mortality with an intrinsic PR interval ≥190 ms.

A prolonged PR interval has been associated with a variety of adverse cardiovascular outcomes such as AF, HF, stroke, dementia, and mortality.^16^, ^17^, ^18^ PR interval prolongation was also found to be correlated with low left atrial voltage and scarring in patients undergoing AF ablation.^19^ Similarly, the current study showed that patients with a longer PR interval have higher incidence of AF. However, in our study we had a benefit of the high accuracy of continuous AF monitoring seen in implanted devices. The incidence of AF was higher in particular in early follow-up in patients with a PR interval longer than 190 ms when compared with shorter PR intervals. However, the incidence of HFH or death manifested a more gradual rise. In a recent randomized trial of patients with sick sinus syndrome and a prolonged PR interval ≥220 ms who required pacemaker implantation, Botto and colleagues^20^ demonstrated that biventricular pacing showed better diastolic function, less left atrial enlargement, lower New York Heart Association functional class, and lower risk of new onset AF compared with conventional dual-chamber pacing with right ventricular pacing avoidance algorithms. Previous longitudinal studies relating the PR interval to prognosis have been almost exclusively restricted to younger men, and our observation further challenges the notion of benign nature of a prolonged PR interval, especially in older patients.^21^

There are several potential explanations for the association of AF with a prolonged PR interval. First, PR prolongation could be a precursor to more severe degrees of conduction block. However, enhanced vagal tone also can prolong the PR interval, although it typically normalizes on long duration follow-up.^22^ Second, PR prolongation could be a marker of other changes in the cardiovascular system that contribute to a worse prognosis or represent advanced structural cardiac abnormalities that may lead to prolongation of the PR interval.^23^ Third, PR interval prolongation might be a sign of intra-atrial or interatrial conduction that has been correlated with the risk of AF.^24^ Fourth, PR interval prolongation results in delayed and ineffective mitral valve closure and diastolic mitral regurgitation, in particular when the PR interval exceeds 230 ms, and it can contribute to increase in left atrial pressure and development of AF.^25^

A prolonged PR interval was found to be associated with development of HF and death in patients with or without coronary artery disease.^3^^,^^26^ There are several potential explanations for this association. First, there is a phenomenon called pseudo-pacemaker syndrome that occurs in the setting of a prolonged PR interval with disruption in atrial emptying and diastolic mitral regurgitation.^25^ In one study, a prolonged PR interval, and in particular a PR interval >300 ms in the setting of sudden increase in AV delay, was associated with significant HF symptoms.^27^ Second, a prolonged PR interval might be secondary to Hisian or infra-Hisian conduction delays, and they are associated with increased mortality in patients with established cardiac disease as a precursor of malignant arrhythmia.^3^ Third, the prolonged PR interval can be a sign of severe cardiac disease and ischemia.^3^^,^^28^ Fourth, in patients with PPM or ICD, prolonged AV delay may increase the right ventricular pacing burden, which has a detrimental effect on cardiac function.^29^ Finally, Magnani and colleagues^30^ reported that prolongation of the PR interval was associated with obesity, waist circumference, and metabolic syndrome, which are also associated with incident HF.

First-degree AV block is defined as a PR interval >200 ms, which is an arbitrary number that is not adjusted for other clinical factors, including age. A wide variety of PR interval cutoffs were studied, and numerous associations with cardiovascular morbidities were found with higher intervals. In the past, the majority of PR interval measurements were based on electrocardiography recordings. In addition, the PR interval cutoff has mostly been treated as a binary value. A PR interval as low as >180 ms was found to be associated with a higher burden of AF.^31^ In addition, Sweeney and colleagues^26^ showed that risk of death or HFH increased by 7.8% for every 10-ms increase in the baseline PR interval above 184 ms.^31^ In our study, we found constant increase in incidence of AF, HFH, or death with increase in PR interval duration. However, this incidence was significantly higher with PR interval ≥190 ms in comparison with lower PR intervals. Given the recent advances in physiological pacing, we believe that the PR interval cutoff is particularly important because it can define a cutoff at which patients might benefit if they receive physiological or resynchronization pacemakers when they have a prolonged PR interval. Botto and colleagues^32^ chose a PR interval ≥220 ms and randomized the patients to cardiac resynchronization therapy vs conventional dual-chamber pacing with minimal right ventricular pacing. Cardiac resynchronization therapy was superior to conventional pacing with regard to diastolic function, left atrial size, and lower incidence of new onset AF. Finally, the HOPE-HF (His Optimized Pacing Evaluated for Heart Failure) trial is currently studying the potential role of His bundle pacemaker optimization of AV conduction in patients with first-degree AV block and HF (ejection fraction ≤40%) and QRS duration <140 ms or right bundle branch block. Patients received His bundle pacing and were entered into 6-month crossover of His pacing vs no pacing (NCT02671903) with the preliminary result showing that the patients with His-optimized pacing had better HF quality of life.^33^

### Study limitations

Similar to other observational and retrospective studies, residual confounding could contribute to the relationship observed. However, the size of the database minimizes the effect of confounders. The restriction of the dataset to a smaller group with more complete data introduces the possibility of bias. In addition, device-based PR interval measurements might be shorter than electrocardiography-based PR interval measurements. However, our pilot study showed that the difference is negligible (unpublished data). Circadian variation of the PR interval also does not affect the outcomes because the transmissions are all done mostly at the same time.^34^ PR interval duration can change over time.^35^ However, longitudinal changes in individual PR interval are typically small (<0.04 seconds).^36^ Finally, our cohort included patients with a higher risk profile, given that they all have PPMs or ICDs. Hence, our findings might not be generalized to healthy individuals. The main strengths of the present study include large sample size, virtually complete outcome ascertainment, and comprehensive adjustment for potential confounding factors. We found an association but not causation, and if anything, these findings are hypothesis generating and should be cautiously interpreted.

## Conclusion

In a large real-world population of patients with implanted devices, PR interval prolongation was significantly associated with increased incidence of AF, HFH, or death. Specifically, there was a significant increase in incidence of AF, HFH, or death with an intrinsic PR interval ≥190 ms. These data suggest that patients might benefit from shortening of the PR interval duration. Therefore, further investigation is recommended to confirm that shortening of the PR internal using physiological cardiac pacing can reduce the cardiovascular morbidities associated with a prolonged PR interval.
